# The Therapeutic Effectiveness Using Fluorescence-Guided Surgery for MRONJ

**DOI:** 10.1155/2022/1650790

**Published:** 2022-09-17

**Authors:** Hongyuan Huang, Ning Zhao, Qingxiang Li, Qiao Qiao, Jianya Zhang, Chuanbin Guo, Yuxing Guo

**Affiliations:** ^1^Department of Oral and Maxillofacial Surgery, Peking University School and Hospital of Stomatology, Beijing 100081, China; ^2^National Clinical Research Center for Oral Diseases, Beijing 100081, China; ^3^National Engineering Laboratory for Digital and Material Technology of Stomatology, Beijing 100081, China; ^4^Beijing Key Laboratory of Digital Stomatology, Peking University School and Hospital of Stomatology, Beijing 100081, China

## Abstract

**Background:**

Long-term application of antiresorptive and/or antiangiogenic agents may cause oral disorders, including medication-related osteonecrosis of the jaw (MRONJ), which remains an incurable disease. Surgical treatment can help alleviate infection of the jaw and block the progress of the disease, but postoperative recurrence is often caused by incomplete resection of necrotic bone during surgery. The traditional method for determining the boundary of necrotic bone resection is primarily based on the color, geology, and microcirculation-based bleeding state according to the bone tissue, which is easily affected by the surgeon's clinical experience and can cause insufficient resection of osteonecrosis bone. Recent studies have proposed using fluorescence technology-assisted necrotic bone resection.

**Objective:**

Systematic literature review was conducted to evaluate the therapeutic effectiveness of fluorescence-guided MRONJ surgery.

**Design:**

PubMed/MedLine, Scopus, and Web of Science databases were searched from inception to February 7, 2022. Randomized controlled trial (RCT) studies were evaluated according to the Cochrane risk of bias tool ROB 2, and non-RCT (N-RCT) studies were evaluated according to the ROBINS-I tool.

**Results:**

A total of 6 articles were included in the systematic review, including 4 N-RCT studies (1 retrospective study and 3 prospective studies) and 2 RCT studies, with 240 patients and 280 MRONJ lesions. The vast majority of studies were with moderate risk of bias, and the quality of the evidence was moderate.

**Conclusion:**

Evidence of moderate strength suggests that fluorescence-assisted techniques effectively determine the bone resection boundaries in MRONJ surgery. However, whether the prognosis of patients treated with fluorescence-guided surgery is significantly better than that of traditional surgery must be proved by randomized controlled studies with larger sample sizes and higher quality.

## 1. Introduction

Antiresorptive and antiangiogenic drugs are widely used in clinical practice and cannot be replaced by other drugs in the short term. Yet, long-term application of antiresorptive and/or antiangiogenic agents may cause oral disorders that involve jaw bone exposure or detectable intraoral or extraoral fistulas, prolonged soft tissue inflammation, and pathological fractures, including medication-related osteonecrosis of the jaw (MRONJ). In 2014, the American Association of Oral and Maxillofacial Surgeons changed the term “Bisphosphonate-Related Osteonecrosis of the Jaws” (BRONJ) to MRONJ, considering that MRONJ is caused by multiple drugs besides bisphosphonates. MRONJ can cause serious functional and masticatory disorders, and its incidence is about 0.01%-0.03% in the osteoporotic population and about 0.1%-3% in tumor patients [1, 2], while the number of new cases is expected to gradually increase [3]. Therefore, exploring effective methods for the treatment of MRONJ is an important issue in clinical research.

At present, there is no definitive treatment modality for MRONJ. Conservative treatments such as mouthwash or antibiotics are often effective in the short-term [4]. Surgical treatment is routinely recommended for patients with advanced stage II-III osteonecrosis [1]. The traditional method of judging the boundary of necrotic bone resection is often determined by the color of the bone resection margin and the status of microcirculation-based spontaneous bleeding to the bone tissue, which is greatly influenced by the surgeon's own experience [5, 6]. False-negative results may result in insufficient sequestrum resection and even disease recurrence [7]. To achieve sufficient resection of necrotic bone, it is often necessary to sacrifice redundant healthy bone tissue during the operation, which, in turn, may cause unexpected complications such as a mandibular fracture or maxillary sinus fistula. Therefore, judging the necrotic bone resection boundaries is a major challenge in the current MRONJ surgical treatment research.

In recent years, clinical reports of fluorescence-guided MRONJ surgery have been increasing. Intraoperative fluorescence help surgeons select enough necrotic bone for resection. The commonly used fluorescence guidance techniques include tetracycline fluorescence and autofluorescence. The difference between the two is that the former requires oral administration of the fluorescent drug doxycycline before surgery to identify the fluorescence and guide the surgery. This article is aimed at systematically reviewing clinical studies using fluorescence-guided surgery technology, evaluate the effectiveness of fluorescence-guided technology in the treatment of MRONJ disease, and summarize the histopathological characteristics of jaw bone tissue with different fluorescence methods.

## 2. Method

### 2.1. Search Strategy

PubMed/MedLine, Scopus, and Web of Science databases were searched from inception to February 7, 2022. The following key terms were used: “medication-related osteonecrosis of the jaws” or “bisphosphonate-related osteonecrosis of the jaw” or “antiresorptive agent-related osteonecrosis of the jaws” jaw” and “fluorescence”. The retrieved literature was published from January 2003 to January 2022.

### 2.2. Selection Criteria

The literature inclusion criteria included the following three items: (1) the study subjects were MRONJ patients undergoing surgical treatment; (2) the study reported the effect of different fluorescence techniques on the mucosal healing rate and jaw bone inflammatory state; (3) the study reported the outcome indicators of mucosal healing and jaw bone inflammatory state.

The exclusion criteria were: (1) data not related to the use of fluorescence-guided technology for MRONJ surgery;(2) laser ablation of sequestrum rather than fluorescence-guided surgery; (3) case reports and case series studies with <10 cases; (4) review articles; (5) nonclinical research (animal experiments).

### 2.3. Data Extraction

After reviewing the titles and abstracts, the literature that met the inclusion criteria was evaluated, and data were extracted. The following data were extracted and recorded: author, year of publication, type of study, number of MRONJ patients, number of MRONJ lesions, MRONJ clinical-stage, patient's underlying disease, history of antiresorptive and/or antiangiogenic drugs application, fluorescence detection technology, intraoperative fluorescence status, histological verification, and clinical outcome.

### 2.4. Risk of Bias and Level of Evidence

Risk of bias (ROB) assessment: randomized controlled trial (RCT) studies were based on the Cochrane risk of bias tool ROB2 [8], and N-RCT studies were based on the ROBINS-I [9]. ROB diagrams were drawn using R software (R Foundation for Statistical Computing, Vienna, Austria) and the robvis package (https://github.com/mcguinlu/robvis). The grading of recommendation, assessment, development, and evaluation (GRADE) instrument were used to assess the quality of evidence for each research [10]. Included studies were evaluated according to their design, study quality, and consistency. Two authors (H.H.Y and Z.N) independently reviewed, extracted data, and performed quality assessments. All disagreements were solved by one oral and maxillofacial surgeon (G.Y.X).

### 2.5. Synthesis and Summary Methods

The primary outcomes were postoperative mucosal healing rate and the remission proportion of inflammatory symptoms in MRONJ patients. Postoperative mucosal healing refers to complete mucosal coverage of the surgical area, no bone exposure, and no intraoral or extraoral fistulas. Inflammation remission refers to the weakening or disappearance of pain and infection symptoms at the original lesion. Secondary outcomes were intraoperative fluorescence status and their corresponding histopathological structures. Study type, number of MRONJ patients, number of MRONJ lesions, MRONJ clinical-stage, patient's underlying disease, history of antiresorptive and/or angiogenic drugs, fluorescence detection technology, intraoperative fluorescence manifestations, histological verification, clinical outcomes, and other information were analyzed in detail.

## 3. Results

### 3.1. Included Literature and Its Characteristics

**Figure 1:**
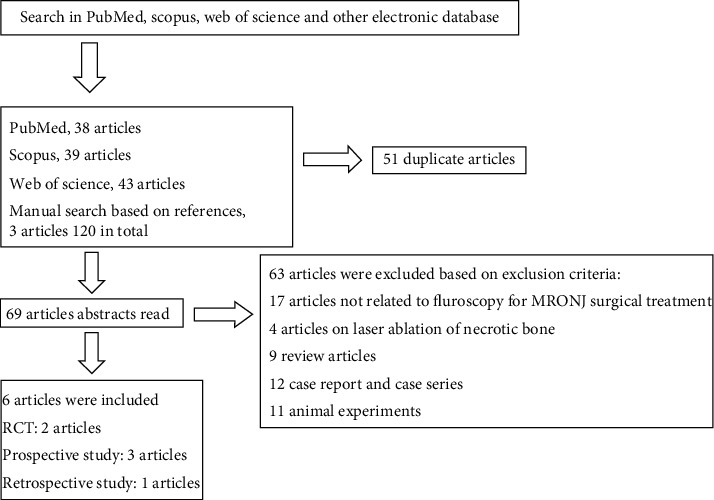
Article inclusion flowchart.

A total of 120 papers were retrieved during the retrieval process, including 38 papers in the PubMed/MedLine database, 39 papers in Scopus, 43 papers in Web of Science, and 3 papers were manually searched according to references. After further review and analysis of the literature, 51 duplicate papers and 63 papers that did not meet the criteria were excluded, and 6 papers were finally included, including 1 retrospective study [11], 3 prospective studies [12–14], and 2 randomized controlled trials [15, 16]. The flow chart of the literature search is shown in Figure 1. Finally, 280 MRONJ lesions in 240 MRONJ patients were included in the study. The number of cases in a single study ranged from 15 to 75, and the number of MRONJ lesions ranged from 20 to 82. Information of study type, number of MRONJ patients, number of MRONJ lesions, MRON clinical-stage, patient's underlying disease, history of antiresorptive and/or angiogenic drugs, fluorescence detection technology, intraoperative fluorescence manifestations, and histological verification are shown in Table 1.

The information of 240 patients included in the 6 studies showed large heterogeneity in primary disease, types of antibone resorption and/or antiangiogenic drugs used, MRONJ clinical-stage, intraoperative fluorescence detection techniques, and effectiveness evaluation; thus, it was impossible to conduct a meta-analysis.

### 3.2. Risk of Bias and Level of Evidence of the Included Literature

**Figure 2:**
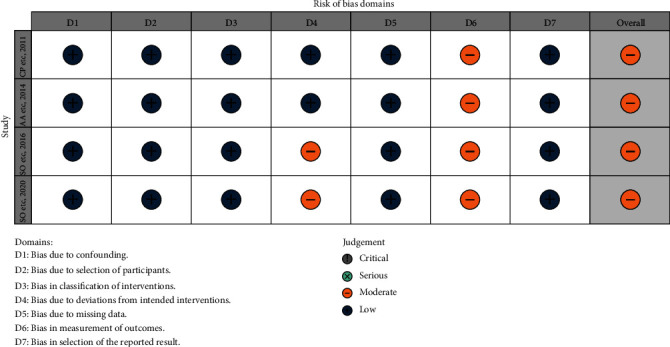
ROB of N-RCT.

**Figure 3:**
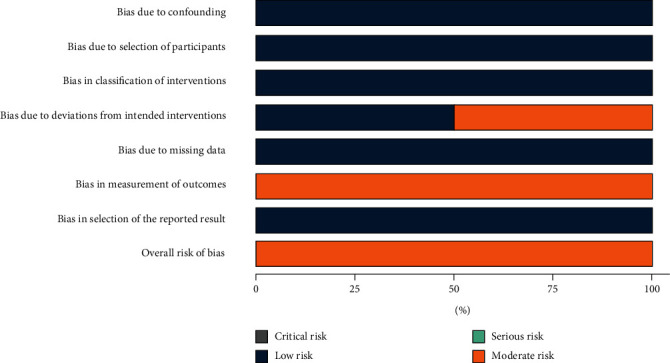
ROB diagrams of N-RCT.

**Figure 4:**
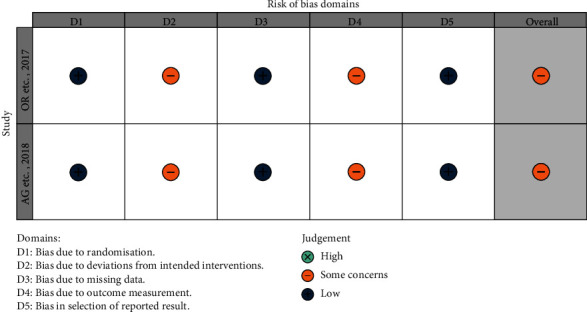
ROB of RCT.

**Figure 5:**
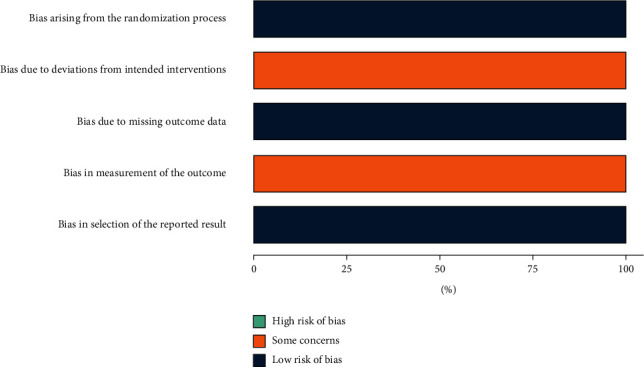
ROB diagrams of RCT.

The risk of bias in the 4 N-RCT studies was moderate. The main source of bias comes from outcome measurements, and none of the four studies were blinded to assess the study outcomes (Figures 2 and 3). In two studies, reoperation was performed in patients who had relapsed after initial surgery, and the source of bias was due to deviations from intended interventions [11, 12]. The risk of bias in the 2 RCTs was moderate, and the source of bias was the fact that the operators of the surgery and the evaluators of the outcome were not blinded (Figures 4 and 5). The quality of evidence for included studies was moderate according to GRADE criteria.

### 3.3. Postoperative Mucosal Healing Rate and Inflammation Remission Rate after Fluorescence-Guided MRONJ Surgery

Intraoperative tetracycline fluorescence was used in 4 of 6 studies [12–14, 16], and the percentage of postoperative mucosal healing ranged from 85% to 91.3%, of which two studies reported the proportion of inflammatory remission of 86.2% and 92.3%, respectively [12, 16]. Intraoperative autofluorescence was applied in 3 out of the 6 studies with postoperative mucosal healing rates ranging from 81.7% to 92.0% [11, 15, 16], of which two studies reported postoperative inflammation remission rates of 94.0% and 95.0%, respectively [15, 16]. One RCT study compared the postoperative mucosal healing rate and postoperative inflammation remission rate after tetracycline fluorescence-guided and autofluorescence-guided necrotic osteotomy, finding no significant difference [16]. Another RCT study compared the postoperative mucosal healing rate and inflammation remission rate between autofluorescence-guided and traditional surgery treatment, and there was no significant difference between the two [15] (Table 2).

### 3.4. Intraoperative Fluorescence Manifestations and Histopathological Features

The fluorescence detection equipment of all 6 studies included VELscope, a portable device for direct visualization of tissue fluorescence. Regardless of tetracycline fluorescence or autofluorescence, normal intraoperative bone showed bright green fluorescence, while necrotic bone showed no or weak fluorescence. One RCT study compared the intraoperative fluorescence performance of tetracycline fluorescence and autofluorescence, and there was no significant difference between the two [16]. Three out of the 6 studies reported histopathological changes in areas of nonfluorescent areas [13, 15, 16]. Inflammation cell infiltration and/or osteonecrosis were confirmed in nonfluorescent areas (Table 3).

## 4. Discussion

Complete removal of necrosis bone is a critical operation procedure in treating MRONJ disease. Pautke et al. suggested that the bone resection boundaries in the bleeding state can not confirm a state of bone health [7]. Therefore, finding an objective, accurate and easy-to-operate method for judging the status of bone resection boundaries has always been an important issue in the surgical management of MRONJ.

Fluorescence technology has already been applied to the auxiliary diagnosis and treatment of oral malignancy and dental hard tissue diseases, such as caries and dental trauma [17, 18]. In recent years, clinical reports of fluorescence-guided technology for the facilitation of bone resection boundaries judgment during MRONJ surgery have been increasing; yet this technology's therapeutic efficiency and histopathological characteristics are still not well understood.

The main clinical manifestations of MRONJ are intraoral or extraoral fistulas, prolonged sequestrum exposure, and inflammatory symptoms such as infection and pain, which seriously affect the patient's quality of life. Therefore, postoperative mucosal healing and remission of inflammatory symptoms are important indicators for evaluating the effectiveness of surgical treatment. The postoperative mucosal healing rate after fluorescence-guided surgery is between 80% and 90%, and the inflammation remission rate is above 90%, suggesting that fluorescence-guided surgery may be used to treat MRONJ effectively. In addition, primary data suggested the clinical therapeutic effectiveness of tetracycline fluorescence and autofluorescence-guided necrotic bone resection in the treatment of MRONJ is similar [16].

The difference between apple-green healthy bone and nonfluorescent necrotic bone under fluorescence guidance is more obvious, and the resection boundary of necrotic bone is more intuitive compared with the traditional subjective judgement (color and structure of the bone tissue and bleeding margins) of the boundary of necrotic bone resection, however, a RCT study indicated that the therapeutic effectiveness of fluorescence-guided surgery was not superior to that of traditional surgery [15]. The postoperative mucosal healing rate and inflammation remission rate were 85.0% and 89.5% in traditional surgery treatment, and 84.2% and 95.0% for autofluorescence-guided surgery treatment. This result may refer to the small study sample and heterogeneous drug administration between the groups,which need to be further explored in larger studies. The fluorescence technology may not be superior to the traditional judgement, but it definitely standardizes the judgment procedure that is heavily dependent on surgeon's own experience.

The earliest research on bone tissue detection by fluorescence technology can be traced back to the 1950s [19, 20]. Tetracycline fluorescence labeling of bone tissue has been used for decades to analyze bone remodeling as well as regeneration processes [21]. Once tetracycline is gradually deposited in healthy bone tissue during bone turnover, the fluorescence signal gradually increases. Under pathological conditions such as inflammation and osteonecrosis, the bone turnover activity is disturbed, resulting in less tetracycline deposition, reducing fluorescence signal. Therefore, differentiating healthy bone from necrotic bone tissue by tetracycline fluorescence is possible.

Before being applied to MRONJ treatment, tetracycline fluorescence technology has been used to assist in the surgical treatment of chronic osteomyelitis, radiation necrosis of the jaw, and other bone inflammatory and necrotic diseases [22, 23]. In 2008, Fleisher first reported using tetracycline fluorescence for BRONJ surgery [24]. Since then, research on tetracycline fluorescence-guided MRONJ surgery has been increasing [25–27]. Doxycycline is the most commonly used drug for tetracycline preoperative labeling. The fluorescent labeling medication prescription is given 7-10 days before surgery, with 100 mg of doxycycline twice a day, orally. The results of tetracycline fluorescence detection shows “apple green” fluorescence in the healthy bone tissue and no or weakened fluorescence signal in necrotic tissue (Table 3). The significant difference in fluorescence signal between healthy bone and necrotic bone provides a criterion basis for bone status identification during MRONJ surgery.

However, a randomized controlled study conducted by Ristow et al. showed no significant difference in the intraoperative fluorescence performance of bone tissue from MRONJ patients detected by the VELscope system regardless of whether the preoperative labeling with tetracyclines was used [16]. There was also no significant difference in the clinical therapeutic effect between the two groups. This suggests that autofluorescence can also be performed without preoperative tetracycline. Furthermore, Ristow et al. conducted a preclinical animal experiment in minipigs to reveal the histopathological mechanism of tetracycline fluorescence and autofluorescence [28]. The fluorescent signal of healthy bone tissue was generated by osteocytes and collagen fibers, while necrotic bone showed no fluorescence due to the destruction of osteocytes and collagen fibers. The so-called tetracycline fluorescence results from the superposition of a small amount of fluorescence generated by the tetracycline and the autofluorescence of bone tissue.

**Figure 6:**
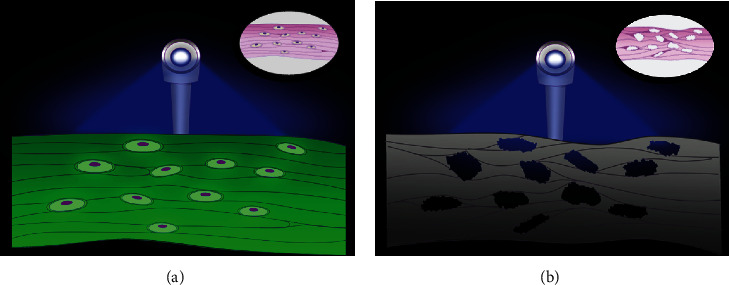
Schematic diagram of fluorescence-guided technology. (a) The fluorescence detection device emits blue fluorescence, and the health bone tissue with osteocytes and collagen fibers appears “apple” green. (b) Necrotic bone showing no or reduced fluorescence. The upper right inset shows a schematic representation of normal bone collagen and osteocytes (a) or necrotic bone and empty lacuna (b).

The intraoperative handheld fluorescence detection device VELscope® (LED Dental, White Rock, British Columbia, Canada) was used in most studies, with or without the introduction of exogenous fluorophores [29, 30]. The device contains a blue fluorescent excitation lamp emitting 400-460 nm wavelength, a green filter, and a camera for capturing and storing fluorescent images. Other fluorescence detection instruments include UV lamps and QLF systems [31, 32]. The fluorescence images observed by the latter two methods differ from the typical “apple green” color of healthy bone under the VELscope system, with no fluorescence of necrotic bone. Under UV light, healthy bone showed blue-violet fluorescence, while necrotic bone showed no fluorescence. In the QLF system, the healthy bone had no fluorescent signal, and the infected and osteolytic areas showed bright red fluorescence, while the abscess and inflammatory cell infiltration areas showed dark-red fluorescence. Although there are differences in the performance of fluorescence images corresponding to the detection instruments, the changes in fluorescence of necrotic bone are based on the destruction of osteocytes and collagen fibers in bone tissue (Figure 6). Therefore, after understanding the mechanism of fluorescence generation in bone tissue, clinicians can choose the fluorescence detection equipment used according to the fluorescence characteristics.

The literature analysis of fluorescence-guided surgery for MRONJ suggests a moderate risk of bias, and the moderate quality of the evidence. The main source of bias is the information bias generated by the lack of blinding in evaluating clinical results. At the same time, the design heterogeneity among studies is large, and there is a lack of RCT studies with a large sample size and high quality. Although there is a significant difference in the fluorescence signal between healthy and necrotic bone, the judgment of intraoperative bone resection boundaries still depends on the surgeon's experience in interpreting the image information of the fluorescent equipment. Future studies should focus on the quantitative study of the correspondence between fluorescence intensity and different histopathological types of bone tissue to assist in the judgment of intraoperative bone resection boundaries.

## 5. Conclusion

The moderate strength of evidence from the above literature review suggests that fluorescence techniques effectively determine intraoperative resection boundaries for MRONJ disease, but it still does not demonstrate a better prognosis than traditional surgery. Therefore, more high-quality RCT studies comparing the prognosis of fluorescence-guided surgery and traditional surgery, and quantitative studies of fluorescence intensity and corresponding histopathological changes are required.

## Figures and Tables

**Table 1:** Basic information of studies reporting on fluorescence-guided surgery for MRONJ.

Author	Study type	No.of cases	No. of lesions	Clinical-stage	Primary disease (no. of cases)	Medication history (no. of cases)	Fluorescence detection technology(no. of cases)	Intraoperative fluorescence	Histological verification
Pautke et al., [14]	N-RCT	15	20	II-III	Malignant tumor (15)	Bisphosphonates (15)	Tetracycline fluorescence, with preoperative doxycycline oral labeling	No or weak fluorescence in necrotic bone	No
Assaf et al., [13]	N-RCT	20	23	I-III	Malignant tumor (18), osteoporosis (2)	Bisphosphonates (20)	Tetracycline fluorescence, with preoperative doxycycline oral labeling	No fluorescence in necrotic bone	Yes
Otto et al., [12]	N-RCT	54	65	0-III	Malignant tumor (45), osteoporosis (9)	Bisphosphonates (47), denosumab (3), bisphosphonates & denosumab (4)	Tetracycline fluorescence, with preoperative doxycycline oral labeling	No or weak fluorescence in necrotic bone	No
Ristow et al., [16]	RCTs	40	51	I - III	Malignant tumor (34), osteoporosis (6)	Bisphosphonates (32), bisphosphonates & denosumab (8)	Tetracycline fluorescence (20), with preoperative tetracycline oral labeling; autofluorescence (20), no oral tetracycline before the operation	No or weak fluorescence in necrotic bone in two types of different fluorescence detection equipment	Yes
Giudice et al., [15]	RCT	36	39	I-III	Malignant tumor (23), osteoporosis (13)	Bisphosphonates (30), denosumab (5), bisphosphonate & denosumab (1)	Autofluorescence (18), no oral tetracycline before operation; traditional surgery (18)	No fluorescence in necrotic bone	Yes
Otto et al., [11]	N -RCT	75	82	0-III	Malignant tumor (65), osteoporosis (10)	Bisphosphonates (51), denosumab (15), bisphosphonates & denosumab (9)	Autofluorescence (18), no oral tetracycline before operation	No fluorescence in necrotic bone	No

**Table 2:** The therapeutic effect of studies reporting on fluorescence-guided surgery for MRONJ.

Authors	Research types of	Surgical procedure	Number of cases	Number of lesions	MRONJ lesion staging				Mucosal healing rate	Inflammation remission rate
					0	I	II	III		
Pautke et al., [14]	N-RCT	Tetracycline fluorescence	15	20	0	0	15	5	85.0%	NA
Assaf et al., [13]	N-RCT	Tetracycline fluorescence	20	23	0	2	10	11	91.3%	NA
Otto et al., [12]	N-RCT	Tetracycline fluorescence	54	65	1	14	42	8	86.2%	86.2%
Ristow et al., [16]	RCTs	Tetracycline fluorescence	20	26	0	3	20	3	88.5%	92.3%
		Autofluorescence	20	25	0	1	21	3	92.0%	94.0%
Giudice et al., [15]	RCTs	Autofluorescence	18	19	0	6	6	7	84.2%	95.0%
		Traditional technique	18	20	0	6	6	8	85.0%	89.5%
Otto et al., [11]	N-RCT	Autofluorescence	75	82	3	3	62	14	81.7%	NA

**Table 3:** Summary of fluorescence manifestations and histological features in articles using fluorescence-guided surgery for MRONJ.

Research	Type of study	Surgical procedure	Fluorescence detection equipment	Intraoperative fluorescence		Histological changes
				Healthy bone	Necrotic bone	
Pautke et al., [14]	N-RCT	Tetracycline fluorescence	VELscope	“Apple green” fluorescence	No or weak fluorescence	NA
Assaf et al., [13]	N-RCT	Tetracycline fluorescence	VELscope	Green fluorescence	No fluorescence	Nonfluorescent areas: Bone resorption destruction, inflammatory cell infiltration, granulation tissue hyperplasia
Otto et al., [12]	N-RCT	Tetracycline fluorescence	VELscope	Green fluorescence	No or weak fluorescence	NA
Ristow et al., [16]	RCTs	Tetracycline fluorescence	VELscope	Green fluorescence	No or weak fluorescence	Nonfluorescent areas are verified as necrotic bone
		Autofluorescence		Green fluorescence	No or weak fluorescence	
Giudice et al., [15]	RCTs	Autofluorescence	VELscope	Green fluorescence	No fluorescence	Nonfluorescent areas are verified as necrotic bone
		Traditional technique	NA	NA		
Otto et al. [11]	N-RCT	Autofluorescence	VELscope	Green fluorescence	No fluorescence	NA
